# 

**Published:** 1874-01

**Authors:** 


### ACKNOWLEDGMENTS.

| | | |
|---|---|---|
| Atlanta Daily Herald | $10 | Per Year. |
| Popular Science, Monthly | 5 | " " |
| Medical Record | 4 | " " |
| Journal of Materia Medica | 1 | " " |
| Medical and Surgical Reporter | 5 | " " |
| The Clinic | 3 | " " |

| | | | |
|---|---|---|---|
| Medical Examiner | 3 | " | " |
| The Western Lancet | — | " | " |
| Virginia Clinical Record | 2 | " | " |
| London Lancet | 5 | " | " |
| Herald of Health | 1.50 | | " |
| Wilson's Herald of Health | 2 | " | " |
| Pacific Medical and Surgical Journal | 5 | " | " |
| Texas Medical Journal | 2.50 | | " |
| The Obstetrical Journal | 5 | " | " |
| American Journal of Obstetrics | 5 | " | " |
| New York Medical Journal | 4 | " | " |
| The Albion | 5 | " | " |
| The Galaxy | 4 | " | " |
| Boston Medical and Surgical Journal | 4 | " | " |
| Boston Journal of Chemistry | 1 | " | " |
| The Plantation | 1.50 | | " |
| Wood's Household Magazine | 1 | " | " |
| Pen and Plow | .50 | | " |
| American Practitioner | 3 | " | " |
| Cincinnati Lancet and Observer | 3 | " | " |
| Druggist's Circular | 1.50 | | " |
| Nashville Jour. Med. and Surgery | 3 | " | " |
| Northwestern Medical and Surgical Journal | — | " | " |
| Baltimore Physician and Surgeon | 1 | " | " |
| Richmond and Louisville Medical Journal | 5 | " | " |
| St. Louis Medical and Surgical Journal | 3 | " | " |
| Detroit Review of Medicine and Pharmacy | 2 | " | " |
| Chicago Medical Journal | 3 | " | " |
| Buffalo Medical and Surgical Journal | 3 | " | " |
| New Orleans Medical and Surgical Journal | 5 | " | " |
| American Journal of Pharmacy | 3 | " | " |
| Medical News and Library | 1 | " | " |

University Record, Sewanee, Tenn.; Methodist Advocate; The Druggist; Calender, University of the South; Calender, University of Michigan; Rockwell and Beard's Electro-Surgery; Toner Lecture, No. 1; Structure Cancerous Tumors, by J. J. Woodward, Assistant Surgeon U.S.A.; Annual Address, Jno. T. Darby, M.D., President of South Carolina Medical Association; Report of Healfh Officer of San Francisco; Report of Managers of Pennsylvania Hospital; Proceedings Nebraska State Medical Society; Catalogue Medical School Harvard University.

